# Editorial: Bioinformatic Tools for the Detection and Identification of Mechanisms in Immune Cells of Neuroimmunological Diseases

**DOI:** 10.3389/fgene.2022.881593

**Published:** 2022-03-21

**Authors:** Sandeep Kumar Dhanda, Tanima Bose

**Affiliations:** ^1^ Department of Oncology, St Jude Children’s Research Hospital, Memphis, TN, United States; ^2^ Institute of Clinical Neuroimmunology, Ludwig Maximilian University of Munich, Munich, Germany

**Keywords:** bioinfomatics, neuroimmunology, computational biology, tools and apllications, immune cells

## Backgrounds

Classically, neuroimmunological diseases include the involvement of both the neuronal and immunological organs and the intertwined relationships between them. Thus, the diseases which have been included in this list of diseases are multiple sclerosis, antibody-related neuronal diseases, and any neuronal diseases where there is an involvement of the immune system and vice versa. This is relatively a new field and people used to doubt the cross-reactivity of both the organs before the landmark publications generated from the field of autoimmune diseases like multiple sclerosis. This is a niche area of research and there has been a need for voice out to facilitate this area of research.

## Noteworthy Computational Methods

**FIGURE 1:**
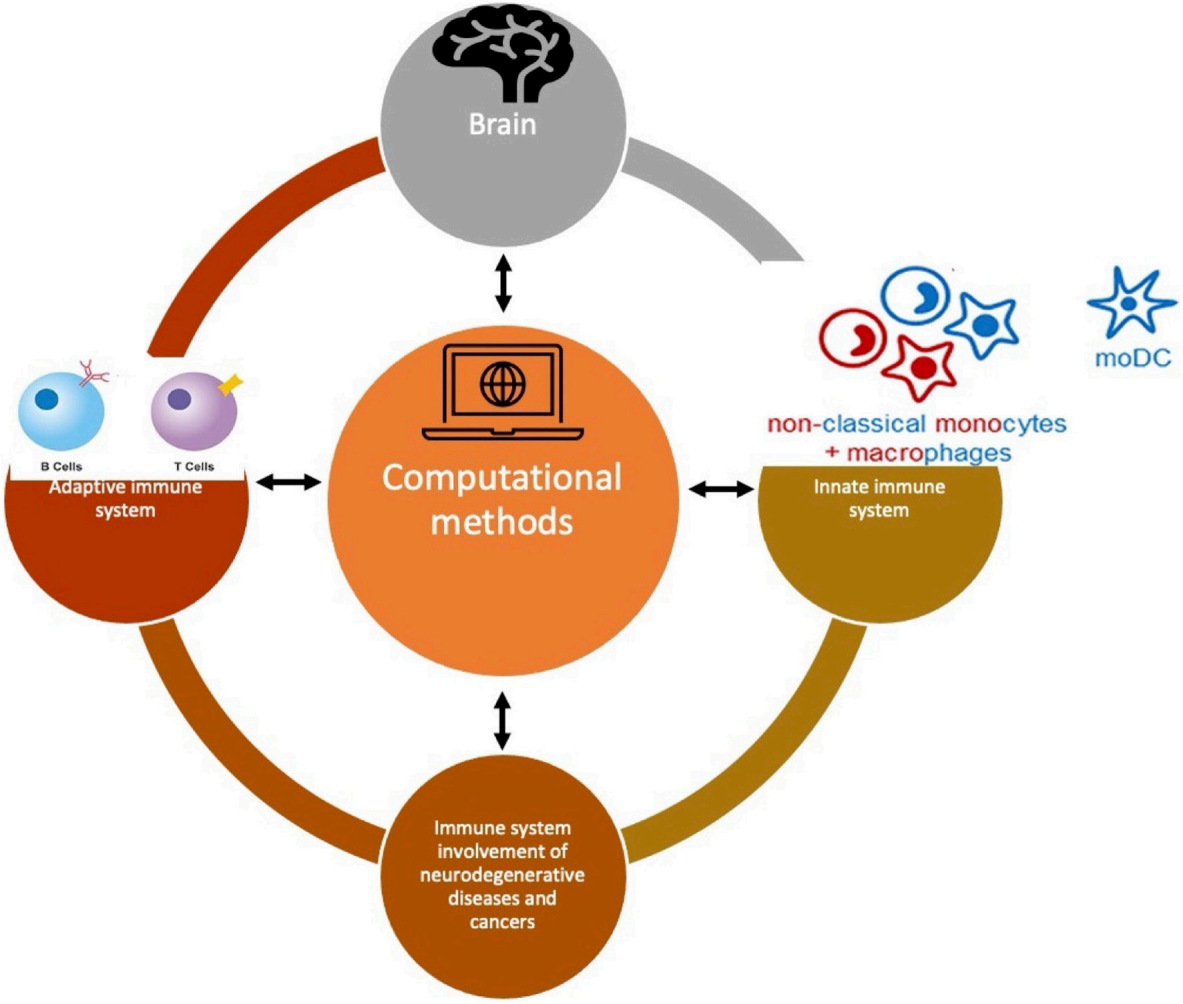
A conceptual figure showing that neuroimmunological diseases are very diverse but connected through common bioinformatics or computational methods to understand their pattern. The different immune cells are illustrated here whereas the brain is shown in general as an organ. All these cells and the organ are connected through computational methods.

The basis for the computational methods is to identify the biomarkers at the transcriptional or translational level and to identify the distinct alterations at the genetic and epigenetic levels (Figure 1). To do so in these cross-organ diseases, several techniques like single-SEQ analysis, proteomic analysis, genome-wide association study (GWAS), gene enrichment, and network analysis have been performed. Another recent trend has been observed in comparing the existing databases on a particular disease like a comparison of TIMER, GEPIA, and LinkedOmics databases for the indication of glioma. Nevertheless, conventional techniques like RT-qPCR, luciferase assay can also stand out if they apply any of the new bioinformatics methods.

## The Idea of this Topic

Considering the undeniable contribution of bioinformatics methods in addressing the critical questions in neuroimmunological diseases, we have proposed this research topic. The research topic is well received in the scientific community, and we received 18 manuscripts for consideration, 8 of them are finally accepted for publication. As this is a niche area, we did not expect a huge number of publications.

## Manuscripts Included

These manuscripts included in the topic cover a wide spectrum of diseases, like multiple sclerosis, Neuro-Behçet Disease, spinal cord injury, Neuromyelitis Optica Spectrum Disorder, Intracerebral Hemorrhage, Alzheimer Disease (AD). However, all of these biologically diverse disease groups were connected through a set of bioinformatics methods applied to delineate the underlying patterns.

In this topic, we acknowledge an insightful piece of work by Liu et al. in addressing the challenge of biomarkers in the diagnosis and treatment of AD. The study offers a model based on seven hub genes and four of them were validated through RT-PCR of serum samples on a small cohort of AD patients and a control group.

Wu et al. performed bioinformatics analysis on proteome data from the plasma and suggested the potential involvement of exosomes in cholesterol metabolism in the acute phase of Spinal cord Injury.

Liu et al. choose computational methods to understand the pathogenesis of Intracerebral hemorrhage. The authors summarized their findings with a set of genes possibly connecting the dots of ferroptosis after cerebral hemorrhage.

Amyotrophic lateral sclerosis (ALS) is another fatal neurodegenerative disorder with limited information on its pathogenesis. Xie et al. addressed this gray area and shed some light on the predictive gene signature to different ALS patients from non-neurological controls.

Further, Alves-Leon et al. reported the first-ever genome-wide investigation on linking neurological manifestation among chikungunya patients. The study presented the data on HLA alleles associated with inflammatory demyelinating diseases (IDD).

Li et al. added another genome-wide association study to delineate the molecular mechanism of Neuromyelitis optica spectrum disorder (NMOSD) and suggested several novel genes from complement, antigen presentation pathways as the central players.

IDD was further explored in the context of multiple sclerosis and neuro-Behçet disease by Maghrebi et al., in an article emphasizing the differential governing mechanisms in these ailments.

Last, but not least, the manuscript in our research topic was contributed by Shan et al. to highlight the transcriptomic profiles and constructed a hub gene network for immune-related genes.

## Conclusion

The combined efforts from these amazing pieces of work put forward strong evidence that bioinformatics methods could help to understand complex mechanisms. However, we, like our contributing authors, are very mindful of interdisciplinary research and are strong proponents of collaborative research. We hope that this topic journal is helpful for other areas of research.

